# Role of oxygen reserve index monitoring in patients undergoing robot-assisted radical prostatectomy: a retrospective study

**DOI:** 10.1007/s00345-024-04938-x

**Published:** 2024-04-13

**Authors:** Jung-Hee Ryu, Young-Tae Jeon, Kyu Man Sim, Soowon Lee, Ah-Young Oh, Chang-Hoon Koo

**Affiliations:** 1 Department of Anesthesiology and Pain Medicine, Seoul National University Bundang Hospital, Seongnam, 13620 Korea; 2 Department of Anesthesiology and Pain Medicine, Seoul National University College of Medicine, Seoul, 03080 Korea

**Keywords:** Trendelenburg position, Hypoxia, Pneumoperitoneum, Prostatectomy

## Abstract

**Purpose:**

Robot-assisted radical prostatectomy (RARP) is a common surgical procedure for the treatment of prostate cancer. Although beneficial, it can lead to intraoperative hypoxia due to high-pressure pneumoperitoneum and Trendelenburg position. This study explored the use of oxygen reserve index (ORi) to monitor and predict hypoxia during RARP.

**Methods:**

A retrospective analysis was conducted on 329 patients who underwent RARP at the Seoul National University Bundang Hospital between July 2021 and March 2023. Various pre- and intraoperative variables were collected, including ORi values. The relationship between ORi values and hypoxia occurrence was assessed using receiver operating characteristic curves and logistic regression analysis.

**Results:**

Intraoperative hypoxia occurred in 18.8% of the patients. The receiver operating characteristic curve showed a satisfactory area under the curve of 0.762, with the ideal ORi cut-off value for predicting hypoxia set at 0.16. Sensitivity and specificity were 64.5% and 75.7%, respectively. An ORi value of < 0.16 and a higher body mass index were identified as independent risk factors of hypoxia during RARP.

**Conclusions:**

ORi monitoring provides a non-invasive approach to predict intraoperative hypoxia during RARP, enabling early management. Additionally, the significant relationship between a higher body mass index and hypoxia underscores the importance of individualized patient assessment.

## Introduction

Robot-assisted radical prostatectomy (RARP) is a widely performed surgery, accounting for 70–85% of prostatectomies performed in the United States [1]. During RARP, a high-pressure pneumoperitoneum is applied, and the patient is placed in the Trendelenburg position [2]. Although this surgical position (high-pressure pneumoperitoneum + Trendelenburg position) has the advantage of improving the surgeon’s visibility and protecting other organs, it can make mechanical ventilation difficult for patients owing to the cephalad movement of the diaphragm [3]. Consequently, hypoxia may occur, leading to tissue hypoxia, hemodynamic instability, and postoperative complications that require immediate intervention [4].

Pulse oximetry is a widely used method for determining oxygen saturation (SpO_2_) and diagnosing hypoxia during surgery [5]. However, it is important to note that SpO_2_ remains almost constant when the partial pressure of oxygen (PaO_2_) exceeds approximately 80 mmHg [6, 7]. This characteristic limits its effectiveness in predicting impending hypoxia, as SpO_2_ values may not decrease significantly until PaO_2_ falls below 80 mmHg. This limitation may require additional monitoring strategies to identify slight changes in oxygenation and offer early warnings of impending hypoxia.

The oxygen reserve index (ORi) (Masimo Corp., Irvine, CA, USA) is a monitoring parameter that can continuously and noninvasively measure oxygen reserves [8]. The index is a dimensionless value ranging from 0.00 to 1.00, corresponding to the arterial partial pressure of oxygen levels ranging from 100 to 200 mmHg [9]. ORi monitoring may aid in detecting hypoxia in advance during ventilatory impairment [8, 9]. Therefore, clinicians may benefit from ORi monitoring for appropriate and prompt management of patients at risk of respiratory compromise.

Our institution uses ORi as a routine monitoring parameter in patients undergoing RARP. This study aimed to analyze the use of ORi in patients undergoing RARP, propose a cut-off value for hypoxia, and investigate its correlation with intraoperative hypoxia.

## Materials and methods

### Ethics statement

This study was approved by the Institutional Review Board of Seoul National University Bundang Hospital (IRB number B-2305-828-101). Because this was a retrospective study, the requirement for informed consent was waived.

### Population

This study included patients who underwent RARP for prostate cancer between July 2021 and March 2023. Patients whose intraoperative ORi data were not recorded, those who did not undergo ORi monitoring during surgery, and those who underwent ORi monitoring after surgical position (high pneumoperitonuem + Trendelenburg position) were excluded.

### Anesthesia and monitoring

Patients were transferred to the operating room after receiving premedication with 0.02 mg/kg of midazolam in the reception area. They were monitored using electrocardiography, pulse oximetry, and non-invasive blood pressure measurements. A sensor (Rainbow® sensor, R1 25L, Revision M, Masimo Corp., Irvine, CA, USA) that can measure ORi and SpO_2_ was applied to the fourth finger and covered with a shield. General anesthesia was maintained with sevoflurane, desflurane, or propofol using target-controlled infusion. Mechanical ventilation was maintained with a fraction of inspired oxygen of 0.5 and a tidal volume of 6–8 ml/kg (ideal body weight). The respiratory rate was adjusted to maintain the end-tidal carbon dioxide at 30–35 mmHg. Positive end-expiratory pressure (PEEP) (0–10 cmH_2_O) was applied at the anesthesiologist’s discretion.

### Surgical procedure

The four-armed da Vinci robot system was used to perform RARP via the transperitoneal approach. Patients were placed in the lithotomy position. The following procedures were performed after docking: (1) bladder detachment; (2) endopelvic fascial incision; (3) dorsal venous complex dissection; (4) bladder neck dissection; (5) dissection of the vas deferens and seminal vesicle; (6) dissection of the posterior, lateral, and apical spaces; (7) posterior reconstruction; and (8) vesicourethral anastomosis and bladder neck reconstruction [10].

### Clinical data collection

We reviewed the electronic medical records and collected pre- and intraoperative data. Preoperative data included age, height, weight, body mass index (BMI), American Society of Anesthesiologists class, smoking history, comorbid pulmonary disease (chronic obstructive pulmonary disease, asthma, tuberculosis, interstitial lung disease, and bronchiectasis), history of lung surgery, and pulmonary function test results. Intraoperative data included the pressure of the pneumoperitoneum, main anesthetics used, PEEP, ORi (recorded 1 min before pneumoperitoneum), SpO_2_, and occurrence of intraoperative hypoxia. Intraoperative hypoxia was defined as SpO_2_ < 95% during the docking of the robotic system, based on the World Health Organization’s guideline and several studies [11–16].

### Statistical analysis

We used R version 3.6.1 (R Foundation for Statistical Computing, Vienna, Austria) with the “pROC” package to draw receiver operating characteristic (ROC) curves and determine the cut-off value [17]. Other statistical analyses were performed using Statistical Package for the Social Sciences version 22 for Windows (SPSS Inc., Chicago, IL, USA). Continuous variables were presented as mean ± standard deviation or median [interquartile range] based on a normal distribution. Categorical variables were presented as numbers (percentages). Univariate and multivariate logistic analyses were performed to identify the potential risk factors for intraoperative hypoxia. Initially, univariate logistic analysis was carried out for all variables, and variables with *P* < 0.1 were selected for multivariate logistic analysis using forward selection. Statistical significance was set at *P* < 0.05.

## Results

**Fig. 1:**
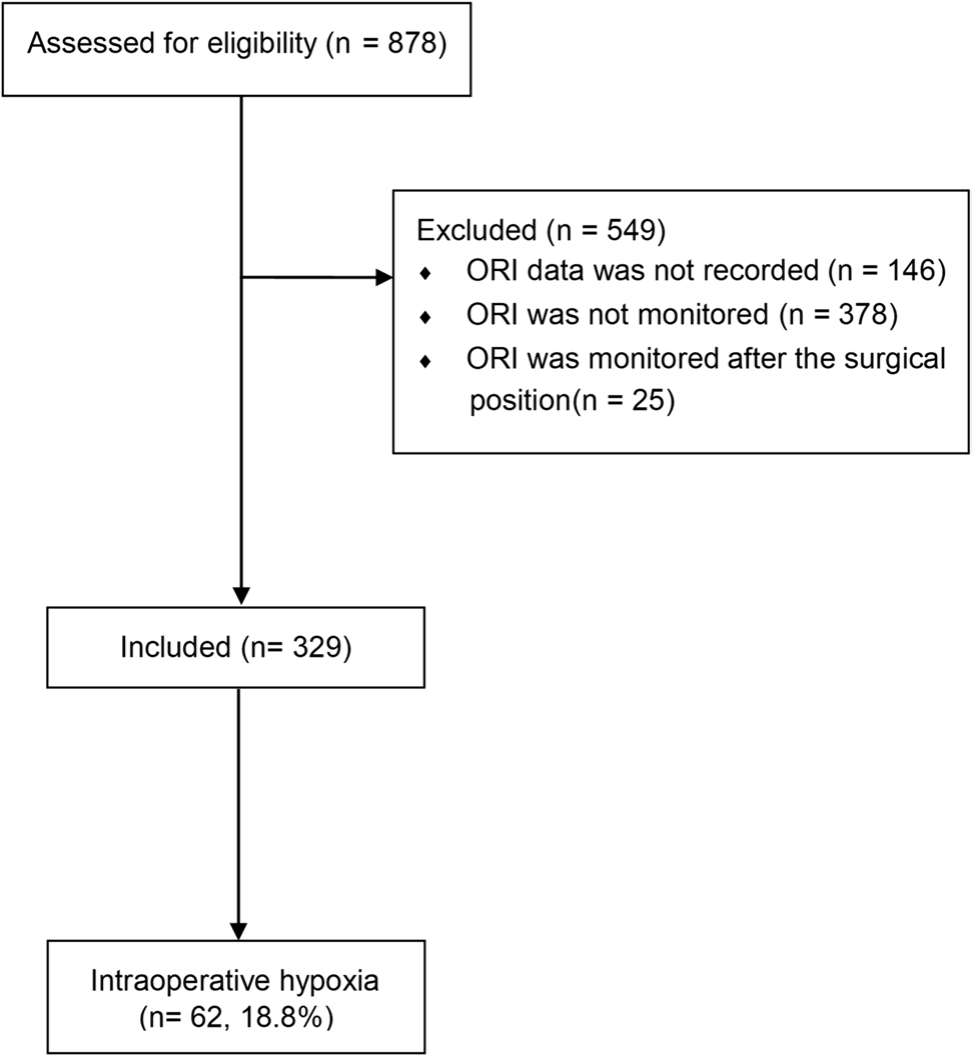
Flow diagram of included patients

A total of 878 patients underwent RARP between July 2021 and March 2023. Of these patients, 146 had no recorded ORi data, 378 were not monitored for ORi during surgery, and 25 were monitored for ORi after surgical position was established (high pneumoperitoneum + Trendelenburg position). Thus, a total of 329 patients were included in the final analysis. Intraoperative hypoxia was identified in 62 patients (18.8%) (Fig. 1). Table 1 summarizes the preoperative and intraoperative variables.

**Table 1:** Clinical characteristics of included patients (*n* = 329)

Variable	
*Preoperative variables*	
Age, year	69 [63, 73]
Height, cm	167.6 ± 5.8
Weight, kg	70.3 ± 9.4
BMI, kg/m^2^	25.0 ± 3.0
*ASA physical status*, *n*	
I	42 (12.8%)
II	243 (73.9%)
III	44 (13.4%)
*Smoking history*	
Smoker, *n*	206 (62.6%)
Pack-year	5 [0, 20]
*Comorbid pulmonary disease*	
Chronic obstructive pulmonary disease	17 (5.25%)
Asthma	3 (0.9%)
Previous TB history	14 (4.3%)
Interstitial lung disease	1 (0.3%)
Bronchiectasis	23 (7.0%)
Previous lung surgery	8 (2.4%)
*Pulmonary function test*	
FVC	3.82 [3.44, 4.24]
FEV1	2.79 [2.44, 3.17]
FEV1/FVC	0.73 [0.68, 0.77]
*Intraoperative variables*	
*Pressure of pneumoperitoneum*, *n*	
10 mmHg	73 (22.2%)
14 mmHg	33 (10.0%)
17 mmHg	223 (67.8%)
*Main anesthetic agent*, *n*	
Desflurane	315 (95.7%)
Sevoflurane	13 (4.0%)
Total intravenous anesthesia	1 (0.3%)
PEEP	5 [5, 5]

Values are expressed as mean ± standard deviation, median [interquartile range], or number (percentage). Abbreviations: *BMI* body mass index, *ASA* American Society of Anesthesiologists, *PEEP* positive end-expiratory pressure, *TB* tuberculosis, *FEV1* forced expiratory volume in one second, *FVC* forced vital capacity

**Fig. 2:**
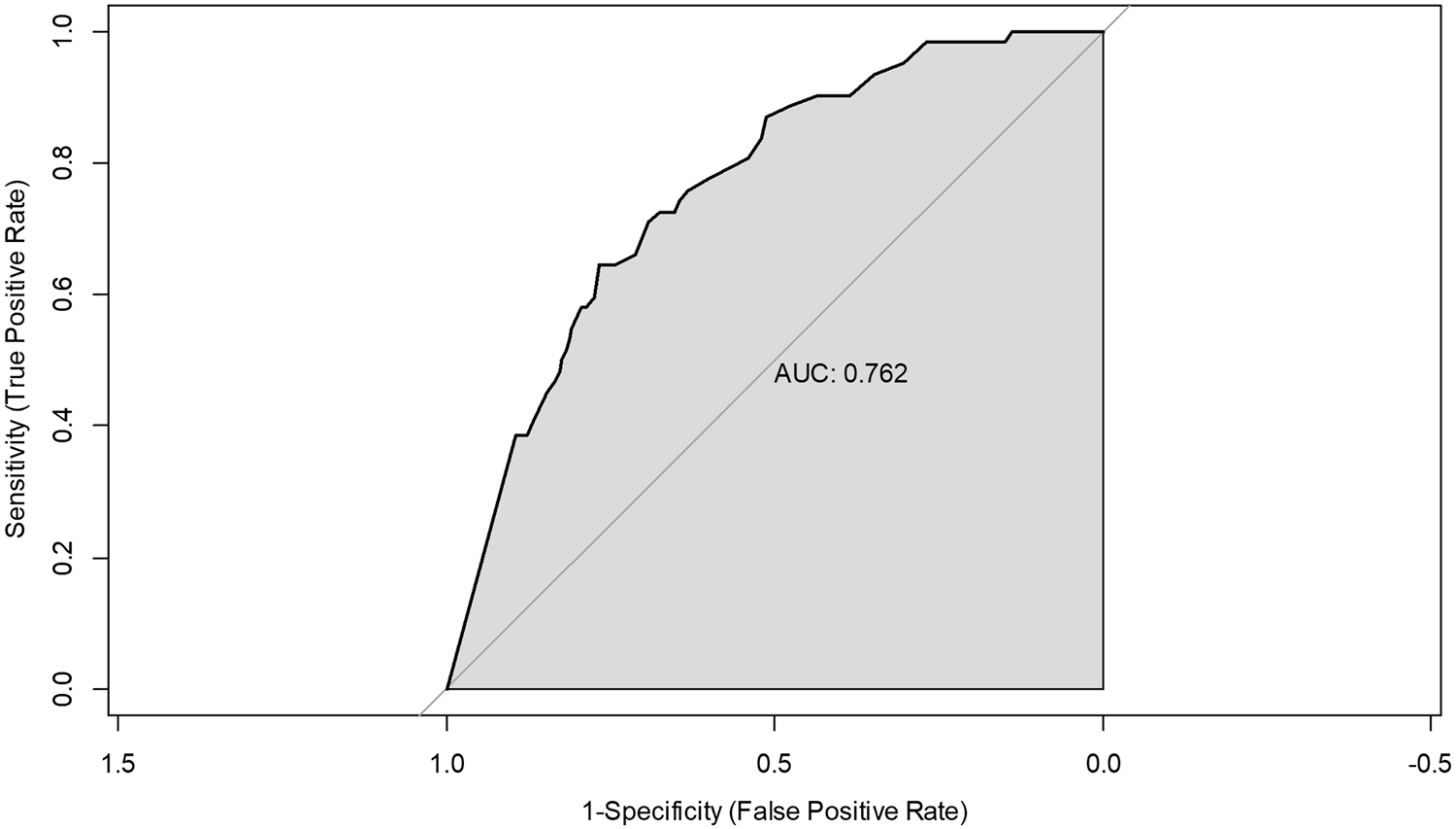
Receiver operating characteristic curve for prediction of intraoperative hypoxia based on the oxygen reserve index

An ROC curve was used to determine the optimal cut-off value for ORi in predicting hypoxia (Fig. 2). The area under the ROC curve was 0.762, which was considered acceptable. The ROC curve showed that 0.16 was the ideal ORi cut-off value for predicting hypoxia. Sensitivity and specificity were 64.5% and 75.7%, respectively.

Table 2 shows the results of univariate and multivariate logistic regression analyses for the occurrence of intraoperative hypoxia. An ORi value of < 0.16 was an independent risk factor for hypoxia during RARP (odds ratio [OR] 3.532, 95% confidence interval [CI] 1.864–6.695, *P* < 0.001). In addition, higher BMI independently increased the occurrence of intraoperative hypoxia (OR 1.348, 95% CI, 1.183–1.535; *P* < 0.001).

**Table 2:** Univariate and multivariate logistic regression analyses of intraoperative hypoxia

Variable	Univariate analysis		Multivariate analysis	
	OR (95% CI)	*P*-value	OR (95% CI)	*P*-value
*Preoperative variables*				
Age, year	1.010 (0.971, 1.049)	0.627		
BMI, kg/m^2^	1.466 (1.296, 1.657)	** < 0.001**	1.348 (1.183, 1.535)	** < 0.001**
*ASA physical status*				
I	(Reference)			
II	1.439 (0.573, 3.614)	0.439		
III	1.543 (0.497, 4.790)	0.453		
*Smoking history*				
Smoker	0.790 (0.450, 1.387)	0.412		
Pack-year	1.007 (0.992, 1.023)	0.366		
*Comorbid pulmonary disease*				
COPD	0.919 (0.256, 3.301)	0.897		
Asthma	0.000	0.999		
Previous TB history	0.708 (0.154, 3.249)	0.657		
ILD	0.000	1.000		
Bronchiectasis	1.576 (0.594, 4.176)	0.361		
Previous lung surgery	2.664 (0.619, 11.461)	0.188		
*Pulmonary function test*				
FVC	0.657 (0.429, 1.006)	0.053		
FEV1	0.824 (0.511, 1.329)	0.427		
FEV1/FVC	27.622 (0.528, 1444.940)	0.100		
*Intraoperative variables*				
*Pressure of pneumoperitoneum*				
10 mmHg	(Reference)			
14 mmHg	0.690 (0.228, 2.091)	0.512		
17 mmHg	0.897 (0.464, 1.735)	0.747		
*Main anesthetic agent*				
Desflurane	(Reference)			
Sevoflurane	0.773 (0.167, 3.578)	0.742		
Total intravenous anesthesia	0.000	1.000		
PEEP	1.062 (0.909, 1.241)	0.446		
*ORi*				
ORi ≥ 0.16	(Reference)		(Reference)	
ORi < 0.16	6.012 (3.323, 10.875)	** < 0.001**	3.532 (1.864, 6.695)	** < 0.001**

Statistically significant *P* values (< 0.05) are highlighted in bold. Abbreviations: *BMI* body mass index, *ASA* American Society of Anesthesiologists, *PEEP* positive end-expiratory pressure, *TB* tuberculosis, *FEV1* forced expiratory volume in one second, *FVC* forced vital capacity, *COPD* chronic obstructive pulmonary disease, *ILD* interstitial lung disease, *ORi* oxygen reserve index

## Discussion

This study aimed to investigate the role of ORi as a predictive measure for intraoperative hypoxia during RARP. We found that an ORi value of < 0.16 and a higher BMI were independent risk factors for intraoperative hypoxia.

Our data indicated that 18.8% of patients undergoing RARP experienced hypoxia during docking of the robotic system. Compared to our findings, a previous study of patients undergoing robot-assisted gynecologic surgery showed a lower incidence of intraoperative hypoxia [18]. Although the surgical position (pneumoperitoneum and Trendelenburg position) was similar to that used in RARP, intraoperative hypoxia occurred in 3.75% of patients. This discrepancy can be explained by the pressure of the pneumoperitoneum applied during robot-assisted surgery. The authors reported that the pressure range was 8–15 mmHg during robot-assisted gynecologic surgery, whereas the pressure was relatively higher (10–17 mmHg) during RARP. The higher pressure used in our study may have influenced more significant physiological changes [19], possibly leading to a higher incidence of intraoperative hypoxia. Another possible reason for this discrepancy could be the definition of hypoxia. Badawy et al. [18] defined hypoxia as SpO_2_ of < 90%, whereas we used a different threshold, which could account for the different rates of hypoxia observed. Patient factors, including age, BMI, and overall health status, may also have contributed to the variation in the occurrence of hypoxia.

Pulse oximetry measures arterial oxygen saturation. Owing to the relationship between SpO_2_ and PaO_2_, SpO_2_ remains relatively stable until PaO_2_ drops below 80 mmHg [6, 7]. Conversely, ORi provides an integrated assessment of both arterial and venous oxygen saturation. Even if SpO_2_ reaches a plateau at PaO_2_ levels above 80 mmHg, variations in venous oxygen saturation may still occur [20]. This principle enables ORi to provide more sensitive and comprehensive information about oxygen status.

In our analysis, the cut-off value of ORi, which helps predict the occurrence of hypoxia, was 0.16. Previous studies have evaluated the ability of ORi to predict hypoxia and suggested a cut-off value for hypoxia occurrence [21, 22]. However, Hille et al. [21] suggested a higher cut-off value for ORi (0.4) to indicate hypoxia during endotracheal intubation in critically ill patients. Applegate et al. [22] found that a decrease in ORi to 0.24 could be a warning sign of lowering PaO_2_ to 100 mmHg. These variations in the cut-off values can be attributed to the different criteria for hypoxia used in the studies (95% vs. 97%). Nevertheless, these findings provide valuable insights into predicting hypoxia using ORi. To the best of our knowledge, this study introduces a novel approach by utilizing ORi, in conjunction with pulse oximetry, to provide a more comprehensive monitoring strategy. This approach aims to detect and warn of impending hypoxia earlier in patients undergoing surgeries that require high pneumoperitoneum and Trendelenburg position, thereby enhancing patient safety and outcomes.

The significant association between ORi and hypoxia observed in our study is consistent with previous research, suggesting that ORi can serve as an early warning signal for hypoxia before it is detected by traditional pulse oximetry. One study explored the use of ORi to measure the oxygen reserves during rapid sequence induction [23]. The authors analyzed data from 16 patients and revealed that ORi could anticipate a decrease in oxygenation approximately 30 s before any notable decline in SpO_2_. They suggested that the incidence of hypoxia during rapid sequence induction can be reduced by ORi monitoring. In another study, including 38 pediatric patients who underwent general anesthesia for ophthalmic artery catheterization, the authors found that ORi values were consistently lower in patients with cardiorespiratory events than those without (0.03 vs. 0.25) [24]. They recommended that clinicians prepare for inadequate ventilation in patients with a low ORi before the procedure. Similarly, observing a low ORi allows for potentially preventive interventions and improved patient safety during RARP.

A higher BMI was independently associated with intraoperative hypoxia. This finding is consistent with the results of a previous study. Kendale et al. [25] conducted a retrospective cohort analysis of 15,238 patients who underwent general anesthesia for noncardiac surgery and found a significant increase in the incidence of intraoperative hypoxia, defined as SpO_2_ < 90%, with increasing BMI.

This study has some limitations. First, the retrospective nature of this study may have introduced a bias. Second, we could not adjust for all potential confounding factors, such as intraoperative fluid management or other comorbidities. Third, more than half of the included patients underwent RARP with a high pneumoperitoneum pressure of 17 mmHg. This factor, along with the variability in standard practices regarding pneumoperitoneum pressure, may limit the generalizability of our findings and underscores the need for further research on the impact of different pneumoperitoneum pressures on clinical outcomes, including the incidence of hypoxia and the predictability of ORi in RARP settings across various surgical practices.

## Conclusions

This study investigated the potential role of ORi as a predictive measure for intraoperative hypoxia during RARP. The findings demonstrated that an ORi value of < 0.16 and a higher BMI were independent risk factors for intraoperative hypoxia. This study emphasizes the potential benefits of ORi monitoring in managing patients undergoing RARP, allowing for immediate intervention and potentially enhancing patient safety. Further prospective studies are needed to assess the applicability of ORi monitoring in procedures beyond RARP and to explore the effectiveness of specific ORi-guided ventilatory management strategies, including a detailed protocol for intervention based on predicted hypoxia.

## Data Availability

The data sets generated and analyzed during the current study are available from the corresponding author on reasonable request.
